# Microencapsulated basil oil (*Ocimum basilicum* Linn.) enhances growth performance, intestinal morphology, and antioxidant capacity of broiler chickens in the tropics

**DOI:** 10.5713/ab.21.0299

**Published:** 2022-01-03

**Authors:** Sureerat Thuekeaw, Kris Angkanaporn, Chackrit Nuengjamnong

**Affiliations:** 1 Department of Animal Husbandry, Faculty of Veterinary Science, Chulalongkorn University, Bangkok 10330, Thailand; 2 Department of Veterinary Physiology, Faculty of Veterinary Science, Chulalongkorn University, Bangkok 10330, Thailand; 3 Food Risk Hub, Research Unit of Chulalongkorn University, Bangkok 10330, Thailand

**Keywords:** Antioxidant Capacity, Basil Oil, Broiler Chicken, Gut Morphology, Microencapsulation, Performance

## Abstract

**Objective:**

Microencapsulation is a technique to improve stability, bioavailability, and controlled release of active ingredients at a target site. This experiment aimed to investigate the effects of microencapsulated basil oil (MBO) on growth performance, apparent ileal digestibility (AID), jejunal histomorphology, bacterial population as well as antioxidant capacity of broiler chickens in a tropical climate.

**Methods:**

A total of 288 one-day-old female broilers (Ross 308) were randomly allocated into 4 groups (6 replicates of 12 birds), based on a completely randomized design. Dietary treatments were as follows: i) basal diet (NC), ii) basal diet with avilamycin at 10 ppm (PC), iii) basal diet with free basil oil (FBO) at 500 ppm, and iv) basal diet with MBO at 500 ppm, respectively.

**Results:**

Dietary supplementation of MBO improved average daily gain, and feed conversion ratio of broilers throughout the 42-d trial period (p<0.05), whereas MBO did not affect average daily feed intake compared with NC group. The broilers fed MBO diet exhibited a greater AID of crude protein and gross energy compared with those in other groups (p<0.05). *Lactobacillus* spp. and *Escherichia coli* populations were not affected by feeding dietary treatments. Both FBO and MBO had positive effects on jejunal villus height (VH), villus height to crypt depth ratio (VH:CD) and villus surface area of broilers compared to NC and PC groups (p<0.05). Superoxide dismutase level in the duodenal mucosa of MBO group was significantly increased (p<0.01), whereas malondialdehyde level was significantly decreased (p<0.01).

**Conclusion:**

Microencapsulation could be considered as a promising driver of the basil oil efficiency, consequently MBO at 500 ppm could be potentially used as a feed additive for improvement of intestinal integrity and nutrient utilization, leading to better performance of broiler chickens.

## INTRODUCTION

High environmental temperature is a principal limiting factor for broiler production, which can cause heat stress (HS), especially in tropical regions. The HS adversely affects physiology status and the productivity of birds [1]. Therefore, the means to increase the bioavailability of certain feed additives is one of the most crucial strategies to boost gastrointestinal function and animal performance [2].

Essential oils (EOs) have already been recognized as a group of functional feed additives with a great benefit to reduce the negative effects of antibiotic growth promoters (AGPs) in animal feed [3–5]. It is well known that AGPs can promote growth performance and heath in animals. Oppositely, the use of AGPs has led to antimicrobial resistance in food chain. Due to pharmacological properties of EOs particularly antimicrobial, antioxidant and anti-inflammatory activities, it could be considered as an alternative to AGPs [6].

Basil oil (BO, *Ocimum basilicum* Linn.) is a natural antioxidant including methyl chavicol (estragole), linalool, eugenol, and methyl cinnamate as main pharmacological constituents [7]. BO containing 925% of methyl chavicol had a better 2, 2-diphenyl-2-picrylhydrazyl radical scavenging activity [8]. In addition to its antioxidant capacity, BO has been also reported for its digestive stimulant, antibacterial, anti-inflammatory activities as well as immunomodulatory effects [9]. However, inconsistent results of BO efficiency in poultry nutrition also have been reported.

The natural compounds of EOs are more fragile and volatile during feed processing, storage conditions (e. g. oxygen, heat, light, and time etc.), and gastrointestinal conditions (e. g. aqueous conditions, endogenous enzymes, and pH) [3,10]. These harsh environments cause the failure of BO in livestock; consequently, an effective protection of these instable ingredients is necessary [11].

Microencapsulation is the formation system of microcapsules that cover the active substance with protective materials. It is used for increasing stability, bioavailability, and the subsequent release of active ingredients at a target site [3,12]. Anionic sodium alginate (SA) and cationic chitosan (CS) have been increasingly used to encapsulate and stabilize EOs for intestinal delivery system due to their biocompatibility, biodegradability, non-toxicity, and the unique pH responsiveness. SA is known to be resistant to acidic gastric juice whereas CS is tolerant of intestinal juice [13–15]. The combination of SA and CS is a good model due to potential formation of the polymer complexes. For example, it was reported that the CS-SA polymer complex increased the stability of glycerol when it passed through the gastric condition and then released in the intestines [14]. However, there have been no in-depth studies of BO in microencapsulated form in broilers. Furthermore, no comparison between the efficacy of microencapsulated BO and its free form has been reported.

In the present work, the novel double-layered microencapsulation was used in manufacturing of microencapsulated basil oil (MBO) which comprised CS as the outer layer and SA as the inner layer. Previously, a CS-SA matrix exhibited an effective delivery in a simulated intestinal model (Supplementary Figure S1). We hypothesized that the MBO would enhance growth performance and gut functions of broiler chickens raised under high temperature environment. Thus, this study was conducted to evaluate the effects of MBO supplementation on productive performance, ileal nutrient digestibility, bacterial population, jejunal histomorphology, and antioxidant capacity of broilers.

## MATERIALS AND METHODS

### Animal care

The experimental protocol was approved by the Animal Care and Ethic Committee of Faculty of Veterinary Science, Chulalongkorn University (Protocol Review No. 1931092).

### Basil oil

Steam - distilled BO from the leaves and flowers was used in this study (Nano Artech Company Limited, Chonburi, Thailand). The gas chromatography-mass spectrometry analysis of the BO composition is shown in Table 1.

### Microencapsulated basil oil

Double - layered microencapsulation was carried out via incorporation of SA and CS. The procedure was composed of three-steps, i.e. the fabrication of SA in the form of porous structure, the absorption of BO in SA porous structure, and the encapsulation of BO-SA porous structure with CS results in the double-layered microcapsule. The MBO with an average size of 1,088±31.0 μm was packed and stored for further feed processing.

### Experimental design, diets, and husbandry

A total of 288 one-day-old female broilers (Ross 308, initial weight = 47.0±0.50 g) were provided by a local hatchery. All chicks were randomly allocated into 4 groups (6 replicates of 12 birds) including i) basal diet without additives as a negative control (NC), ii) basal diet with avilamycin at 10 ppm as a positive control (PC), iii) basal diet with free basil oil (FBO) at 500 ppm, and iv) basal diet with MBO at 500 ppm, respectively. FBO was prepared by spraying BO onto silicon dioxide (SiO2) as a carrier. A feeding program consisted of 3 phases: a crumbled starter diet (days 1 to 21), a pelleted grower diet (days 22 to 35) and a pelleted finisher diet (days 36 to 42), respectively. The basal diets were formulated with corn and soybean meal to meet or exceed the nutrient requirements for Ross 308 broiler chickens [16]. All diets were pelleted at a steam conditioning temperature of 80°C for 40 s. The diet and clean tap water were provided *ad libitum* throughout the experiment. The feed ingredients and chemical compositions of the diets are shown in Table 2. The broiler chickens were reared on floor pens (100×150 cm) in a conventional house at the Student training center, Department of Animal husbandry, Faculty of Veterinary Science, Chulalongkorn University, Nakhon Pathom, Thailand. A lighting program was 22 h light during the first 14 days under incandescent lamp and then 12 h light and 12 h darkness from d 15 to the end of the trial. The indoor temperature ranged from 25°C to 28°C and 33°C to 35°C in the morning and the afternoon, respectively. Relative humidity (%, RH) was recorded in the range of 84% to 87%.

### Growth performance

Growth performance was measured for days 1 to 21, 22 to 42, and 1 to 42. Body weight (BW) and feed intake (FI) were recorded to calculate average daily feed intake (ADFI), and average daily gain (ADG). The feed conversion ratio (FCR) was also calculated by dividing ADFI with respective ADG. Mortality rates were recorded and calculated throughout the experiment.

### Sample collection and analysis

#### Apparent ileal digestibility

The broiler chickens (3 chicks from each replicate, 18 chicks per treatment) were randomly selected, tagged using plastic band and provided 20 g/kg diet of acid-insoluble ash (AIA) as an indigestible marker in two collection periods (days 18 to 21 and days 39 to 42). At the end of two collection periods, these birds were euthanized using CO_2_ inhalation. Then, ileal digesta samples (2 to 3 g) were collected and pooled for each replicate pen. Diets and ileal contents were dried to determine dry matter (DM), crude protein (CP), ether extract (EE), crude fiber (CF), ash, and nitrogen free extract (NFE), according to the association of official analytical chemists [17]. Gross energy (GE) was analyzed using a bomb calorimeter with benzoic acid as a reference standard (Mode AC-500; Leco, St. Joseph, MI, USA). AIA was also determined using the method of Vogtmann et al [18]. The apparent ileal digestibility (AID) was calculated using the following Eq. 1.


Eq. 1 $$\text{AID }(\%)=100-[({\text{AIA}}_{\text{diet}}/{\text{AIA}}_{\text{digesta}})\times ({\text{Nutrient}}_{\text{digesta}}/{\text{Nutrient}}_{\text{diet}})]\times 100$$

Where AIA_diet_ is the AIA concentration in the diet, AIA_digesta_ is the AIA concentration in the ileal digesta, Nutrient_digesta_ is the nutrient concentrations in the ileal digesta and Nutrient_diet_ is the nutrient concentration in the diet.

#### Jejunal histomorphology

The segment of mid-jejunum (~2 to 3 cm) was taken from euthanized 3 birds per replicate on days 21 and 42. The whole jejunum was located between endpoint of the duodenal loop and the Meckel’s diverticulum. The intestinal tissue was flushed and immediately fixed in 10% formaldehyde solution, followed by tacking in paraffin wax [6]. Afterward, fixed intestinal samples were placed on a glass slide and then stained using hematoxylin and eosin. Villus height (VH), villus width (VW) and crypt depth (CD) were measured using a light microscope at 100× magnification (Mode BX5; Olympus, Tokyo, Japan). These data were calculated for the villus height to crypt depth (VH:CD) ratio and villus surface area (VSA). VSA was calculated using the following Eq. 2.


Eq. 2 $$\text{Villus surface area }(\text{VSA},{\text{mm}}^{2})=2\pi \times (\text{Average villus width}/2)\times \text{Villus height}$$

#### Bacterial population

Enumeration of bacterial population was carried out by collecting fresh ceco-colon contents (1 to 2 g) from 3 birds per replicate on days 21 and 42 [19]. *Lactobacillus* spp. was determined by colony forming unit (CFU) quantification technique. The samples were pooled, then diluted with 9 mL of sterile peptone water and mixed on a vortex stirrer. Then, each diluted sample was anaerobically cultured on de Man, Rogosa, Sharpe agar (MRS agar) and incubated at 37°C for 48 h. The colonies of bacterial population are expressed as log_10_ CFUs per gram of fresh content. The enumeration of *Escherichia coli* (*E. coli*) was determined by the most probable number (MPN) technique [20]. Three series of dilutions contained nutrient broth and different amounts of sample (0.1, 1.0, and 10 mL). All tubes were incubated at 37°C for 24 h. Then, the positive tubes were used to confirm the growth of *E. coli*. The obtained results are expressed as MPN per gram of fresh content.

#### Antioxidant capacity

Duodenal mucosa (~2 to 3 g) from 3 birds per replicate on days 21 and 42 was scrapped using a glass microscope slide, pooled, and immediately stored at −20°C. Then, the representative sample was homogenized with 0.05 M phosphate buffer solution at pH 7.4. The homogenates were centrifuged for 15 min at 1,500×g (4°C). The supernatant was then used for analyses of superoxide dismutase (SOD) and malondialdehyde (MDA) contents, which were used as the indexes to monitor the antioxidant capacity.

The qualitative analysis of SOD was performed as follows [21]. The change of chemiluminescence of luminol which produced superoxide radical by the reaction of xanthine and xanthine oxidase was observed using a UV-VIS spectrophotometer (Evolution 201 series; Thermo scientific, Waltham, MA, USA) at 560 nm. The reaction tube contained the supernatant of duodenal mucosa (50 μL), 0.25 mmol/L xanthine, 1.0 mmol/L luminol and 0.10 mmol/L ethylenediaminetetraacetic acid in a 50 mmol/L carbonate buffer. The SOD activity was expressed as unit/mg protein of intestinal mucosa. The activity of SOD causing a 50% inhibition of the chemiluminescence was defined as 0.01 unit. The MDA concentration was measured by the thiobarbituric acid reactive substances method [22]. One milliliter of supernatant was added and mixed with 1 mL of thiobarbituric acid. The mixture was heated in a water bath at 100°C for 30 min and then cooled down. The absorbance was measured at 535 nm using a UV-VIS spectrophotometer. Protein was determined using bovine serum albumin as a standard. All analyses were performed in triplicates.

### Statistical analysis

All data were statistically analyzed by one-way analysis of variance using SAS statistical software [23]. Differences between mean values of the treatments were determined using post-hoc Tukey’s HSD test. The pen mean served as the experimental unit, according to the following model:


$${\text{Y}}_{\text{ij}}=\mu +{\text{T}}_{\text{i}}+{\text{e}}_{\text{ij}}$$

where Y_ij_ is the dependent variable observation, μ is the overall mean, T_i_ is the effect of the treatment, and e_ij_ is the random error. A p-value ≤0.05 was considered as a significant effect of the treatment. Results are presented as mean and standard error of mean.

## RESULTS

### Growth performance

The influence of experimental diets on growth performance of broiler chickens are summarized in Table 3. Final BW of broilers in NC was significantly lower than that in other groups (p<0.01). Experimental diets had no significant effect on ADFI throughout the study. During day 1 to 21, the supplementation of FBO and MBO at 500 ppm and PC tended to increase ADG (p = 0.059), but no differences were found during days 22 to 42 (p = 0.131). Overall, ADG in broilers fed with PC, FBO, and MBO was improved compared with that in NC (p<0.01), but there were no significant differences among 3 treatments. Broilers fed with PC, FBO, and MBO diets had a lower FCR than those fed with the NC diet (p< 0.01) during days 1 to 21 and tended to improve FCR than those fed with NC diet (p = 0.058) during day 22 to 42. Overall, broilers receiving MBO, and PC diets exhibited a lower FCR than those receiving NC (p = 0.008), but there were no significant differences between NC and FBO diets. The overall mortality rate was not affected by treatments (p = 0.862).

### Apparent ileal digestibility

The influence of MBO supplementation on AID is shown in Table 4. On day 21, PC, FBO, and MBO exhibited a significant positive effect on the AID of DM and NFE (p<0.05) compared to NC. The AID of CP was increased by PC and MBO diets than NC diet (p = 0.002), but there were no significant differences between NC and FBO diets. Broilers fed with MBO diet improved the AID of EE (p = 0.032) and GE (p<0.01) than those in the fed with NC diet. The AID of CF and ash was not influenced by experimental diets on day 21, but the digestibility of CF was greater in PC, FBO, and MBO diets than in NC diet (p<0.05) on day 42. The AID of CP and EE with MBO supplementation was significantly increased (p<0.05) compared to that of NC group on day 42. However, there was no significant difference among diets supplemented with additives. The digestibility of DM, ash and NFE was not influenced by experimental diets on day 42. Broilers fed with MBO had greater AID of CP than those fed with FBO on day 21 (p<0.05), whereas MBO supplementation had more beneficial effect on GE digestibility (p<0.05) than PC groups on day 42.

### Jejunal histomorphology

The morphological changes of the mid-jejunum in broilers are presented in Table 5. Jejunal VH was improved by MBO in comparison to NC and PC treatments on day 21 (p = 0.036). However, there was no statistical difference between FBO and MBO diets. On day 42, FBO and MBO groups had higher VH than NC and PC groups (p<0.01). Jejunal CD and VW were not significantly affected by dietary treatments. On day 21, broilers fed with MBO diet had greater VH:CD value than those fed with NC, PC and FBO diets (p = 0.012). Moreover, it had a similar trend (p = 0.022) on day 42, except that there were no significant differences between MBO and FBO treatments. The MBO diet increased VSA of jejunum compared with the NC and PC diets (p<0.05) on day 21 and day 42. VSA in FBO and MBO groups did not differ significantly on day 21, but the difference was observed on day 42 (p<0.01).

### Bacterial population

The effects of dietary MBO on bacterial population in broilers are shown in Table 6. Experimental diets affected neither the population of *E. coli* nor *Lactobacillus* spp. in ceco-colon in any phases.

### Antioxidant capacity

The results of antioxidant capacity are illustrated in Table 7. On day 21, broilers in MBO group significantly increased SOD of duodenal mucosa compared with those in NC and PC groups (p<0.01). However, there was no significant difference in SOD of duodenal mucosa between FBO and MBO groups. The NC group has the least activity of SOD. On day 42, broilers in MBO group showed the highest SOD activity (220 U/mg protein) in comparison with other groups (p<0.01). The highest value of MDA was found in broilers receiving NC diet for days 21 and 42. On the other hand, a significant reduction (p<0.01) in MDA level was found in duodenal mucosa of birds receiving MBO diet throughout the experiment.

## DISCUSSION

This experiment was performed in a conventional house during the summer period. When broilers are raised under high temperature environment, they usually undergo chronic HS (over 30°C for 10 h/d and for 20 consecutive days), leading to lower performance [1]. In this study, the resistance to chronic HS in broilers via the development of natural antioxidants with microencapsulation was examined. Microencapsulation has been known as a promising process for encouraging the performance of additives in a wide range of industries. This technique can be used to improve thermostability and oxidative stability, to extend shelf life, to cover undesirable tastes and to reduce the irritation on users in addition to improving the release properties [10,12]. In general, the small intestine is a target site for the evaluation of EOs properties. The changes in intestinal physiology are the main criteria for consideration of gut morphological development and function. The result indicated that broilers in NC had the shortest VH and the lowest VH:CD and VSA, whereas a positive impact was seen in the MBO group. This evidence indicates the vital role of MBO to improve the intestinal morphology. Consistent with our results, Khattak et al [24] found that inclusion of EOs (basil, caraway, laurel, lemon, oregano, sage, tea, and thyme) increased the length of villi and their absorptive surface areas of broilers, thus increasing body weight gain (BWG). MBO had slightly more positive effects on VH, VH:CD, and VSA of jejunum than FBO. Previous results indicated there was no effect in histomorphology and microbiota in broilers between free and encapsulated forms of garlic and *Phyllanthus niruri* L extracts [25]. The improvement of jejunal histomorphology in this study could be elucidated by the action of methyl chavicol (phenylpropene) which is the dominant constituent of BO [7]. It acts as free radical scavenger by donating its phenolic hydroxyl (OH) groups to reactive oxygen species (ROS) thereby potentially reducing oxidative stress, tissue damage, and lipid peroxidation. BO loaded with nystatin represented a high ability to remove free radicals [26]. As it is known, the increase in villi height is associated with large absorptive area and nutrient uptake efficiency, which leads to increased BW gain. Likewise, the improvement of VH:CD involves the turnover rate of villi which reduced energy for maintaining the gut function and increased energy reserves. In addition, the integrity of intestinal tissue has been reported to be directly linked with the improvement in brush border enzyme expression and nutrient transport systems. The positive effect of MBO on VH possibly reflected an increased concentration of BO in the jejunum. As a consequence, the VH:CD and VSA in MBO were also increased. In the same way, the supplementation of microencapsulated phytogenic at 100 mg/kg with pure curcumin at 50.0 mg/kg increased VH:CD ration in broilers [27].

Exposure of broilers to high ambient temperature may trigger over ROS production, which causes oxidative stress. As mentioned, animals lost balance of antioxidant defense system. SOD is a key enzyme in the antioxidant defense system and MDA is a marker of lipid peroxidation. Our study demonstrated a strong positive impact of MBO on SOD activity and MDA content in duodenal mucosa. The current findings were comparable with the study of Hashemipour et al [28] who described that thymol and carvacrol exhibited greater activity of antioxidant enzyme in thigh muscle, serum, and liver of broilers. In the grower-finisher period, the difference of SOD activity among treatments was clearly observed. This might be due to an increase in metabolic rate and high environmental temperature. Excessive ROS may cause impaired defense mechanism such as inactivation of antioxidant enzymes. Besides, plant derivatives may enhance antioxidant enzymes via regulation of gene expression [29]. The positive effect on MDA content is related to the mechanism of methyl chavicol as previously described in intestinal parameters [7,26]. It was reported that ginger extract decreased MDA in broilers exposed to HS [30]. Alleviation of intestinal inflammation by increasing antioxidant capacity has been reported previously [19]. As compared with those in the FBO group, the MBO with SA and CS decreased the MDA levels and increased SOD activity, especially in the last period of age. Microencapsulation could function as a protective means in antioxidant activity of BO. Similarly, CS particles sustained the antioxidant activity of clove EO better than its free form [31].

Antimicrobial activity of EOs is linked to their functional groups of bioactive constituents [5,11]. EOs could destroy bacterial cell wall as the main target site, leading to damaging bacteria and eventually the bacterial death. Methyl chavicol and linalool showed strong antibacterial activity based on *in vitro* study [32] but no significant effect was found in this experiment. The study of Wang et al. [33] suggested that a combination of encapsulated thymol (10%) and sorbic acid (20%) plus fumaric acid (20%) increased population of *Bifidobacterium* in laying hens. Broilers in this experiment had no response on bacterial population possibly due to relatively low percentage of linalool, absence of eugenol and synergistic action between them as compared to previous report by Pandey et al [34]. Koroch et al [35] found that sweet basil with linalool (54%) - eugenol (19%) chemotype showed the high antimicrobial activity. However, the mode of action of those constituents is still not fully understood and the studies of their effects in poultry production are limited. It is difficult to directly compare these results with those previous reports because of high variation of bioactive compounds and optimal dosage in each product, bacterial profile and physiological functions of the host animals [4]. In a preliminary study, BO with high methyl chavicol had great antioxidant activity and a weaker antibacterial activity (Supplementary Table S1). Due to the property of BO, the combination of BO and other antimicrobial additives such as organic acids may enhance growth performance in broilers. The improvement in above-mentioned parameters all associated with nutrient digestibility and growth performance. MBO exerted greater AID of CP and GE by 5.01% and 2.13% for 21 day and by 3.17% and 3.83% for day 42 in comparison to FBO, respectively. Similarly, the adding of thyme and star anise encouraged apparent total tract digestibility of GE and nutrients in broilers orally challenged with *Clostridium perfringens* [36]. Carvacrol, thymol, and limonene at 100 ppm in encapsulated form improved AID of nutrients and growth performance in broilers [37]. This is possibly due to the stimulated secretion of bile, mucus, and endogenous enzymes (trypsin, chymotrypsin, lipase, and amylase) in pancreas and intestinal wall [38]. The high CP digestibility in MBO group may enhance bioavailable amino acids and cell proliferation, resulting in increased intestinal integrity. The AID result was positively linked to growth response. Under high ambient temperature, it was found that broilers had lower FI and BW than the standard performance of Ross 308. However, MBO was effective in enhancing ADG and FCR by 9.43% and 8.85%, respectively compared to NC in overall period. The supplementation of thyme EO resulted in an improved growth performance of heat-stressed broilers [5,39].

These results indicate that dietary manipulation with MBO might ameliorate the adverse impacts on growth performance of chickens raised under tropical conditions. In case of encapsulated forms, turmeric extract nanoparticle had a significant influence on BWG, but no significant influence on FI and FCR in broilers [40]. Thyme EO in chitosan nanoparticles also improved BWG and FCR [41]. Nevertheless, the different outputs of growth performance may be due to variable compositions and dosages of active ingredients, basal diets, animal condition, and experimental condition [4]. The bioavailability of FBO and MBO after passing through feed processing and the digestive system was described as follows. The FBO was partially lost in feed processing due to evaporation and degradation during hydrothermal processing whereas the unique characteristic of CS-SA matrix improved the stability of MBO. The FBO entering the proventriculus may stimulate the secretion of hydrochloric acid (HCl) and pepsin, leading to increased digestibility. However, the movement of FBO into the intestine was relatively limited because of its poor solubility in water. Morphological changes of MBO were found in proventriculus as follows. The outer layer of MBO (CS) was swollen because of the gastric pH environment. This might cause the protonation of amine groups of CS, resulting in the chain expansion and absorption of a large amount of water. As a result, CS was gradually solubilized. The inner layer of MBO (SA) maintained in acidic pH and assisted BO to withstand endogenous enzymes. The SA facilitated the delivery of BO because of the hydrophilic properties. The remaining FBO was formed as micelles by bile salts thereby enhancing their properties at the end of duodenum and jejunum. Similar to FBO, the SA of MBO was initially dissolved at pH>5 due to the protonation of the ionic carboxylate groups, leading to the diffusion of BO into lower part of intestine. Consequently, MBO may maintain the most possible amount of bioactive compound, thus facilitating intestinal function. According to Omonijo et al [10] who reported that alginate microcapsules of thymol showed a delayed release in stomach and subsequently functioned in intestine. The release mechanism, as previously described, was dependent on type of polymer and its characteristic, cross-linking degree, environmental conditions, and incubation time. Nevertheless, there are both advantages and disadvantages of two forms at different parts of gastrointestinal tract. As a result, MBO and FBO did not have any difference in growth performance. Another possibility is that the optimal efficiency of BO on overall responses may be less than 500 ppm. Future work should consider the dose reduction of MBO to prove the proposed objectives and the maximum economic benefit. Additionally, methyl chavicol and linalool are a relatively new group of feed additives and their applications with microencapsulation should be investigated. The potential of BO in this study on growth performance was found to be equally effective in feed as avilamycin.

## CONCLUSION

The MBO containing methyl chavicol and linalool at 500 ppm has a potential to improve nutrient digestibility and growth performance through modifying jejunal histomorphology and antioxidant capacity of broilers in the tropical climate. Compared with FBO, the MBO promoted VH:CD, VSA, CP, and GE digestibility as well as modulated antioxidant capacity. Moreover, the supplementation of MBO or FBO could be considered as an alternative to AGPs.

## Figures and Tables

**Table 1:** Chemical composition of basil oil (*Ocimum basilicum* Linn.)

No.	Compound	R.I (s)	%
1	Alpha-pinene	396	0.070
2	Beta-pinene	471	0.050
3	6-methyl-5-hepten-2-one	509	0.090
4	Eucalyptol	557	0.150
5	Beta-ocimene Y	591	0.130
6	trans-linalool oxide	625	0.110
7	Cis-linalool oxide	646	0.170
8	Linalool	685	22.1
9	Menthol	764	0.340
10	Methyl chavicol	790	65.9
11	Z-citral	814	1.50
12	E-citral	843	1.53
13	Trans-caryophyllene	967	0.750
14	Alpha-bergamotene	977	1.39
15	Beta-farnesene	997	0.870
16	Germacrene D	1,021	0.780
17	Beta-bisabolene	1,040	0.240
18	Delta-cadinene	1,054	0.080
19	Cis-alpha-bisabolene	1,067	3.83
	Total		100

R.I. (s), retention time (unit: second).

**Table 2:** Ingredients and calculated composition of the basal diet (as-fed basis)

Item	Feeding phase		

	Starter	Grower	Finisher
Ingredient (%)			
Corn	56.0	61.3	63.6
Soybean meal	35.0	30.2	27.8
Soybean oil	4.60	4.50	4.86
MDCP	1.81	1.58	1.49
Limestone	1.11	0.99	0.94
Salt	0.25	0.25	0.25
Vitamin and mineral premix^1)^	0.20	0.20	0.20
DL-Methionine	0.28	0.26	0.25
L-Lysine HCl	0.17	0.16	0.16
L-Threonine	0.09	0.06	0.05
L-Isoleucine	0.02	0.01	0.00
Sodium bicarbonate	0.19	0.19	0.15
Choline Chloride 60%	0.07	0.08	0.08
Antimold (Propimpex)	0.20	0.20	0.20
Salinomycin (Sacox)	0.05	0.05	0.00
Total	100	100	100
Calculated nutrient composition (%)			
Dry matter	87.9	87.8	87.8
Metabolizable energy (kcal/kg)	3,100	3,150	3,200
Crude protein	22.0	20.0	19.0
Ether extract	7.23	7.25	7.65
Crude fiber	2.95	2.83	2.76
Lysine	1.32	1.18	1.11
Methionine	0.62	0.57	0.55
Met+Cys	0.98	0.91	0.88
Calcium	0.90	0.80	0.76
Total phosphorus	0.77	0.70	0.67
Available phosphorus	0.45	0.40	0.38

MDCP, mono-dicalcium phosphate.

1) Premix composition per kg of feed: vitamin D, 2,400 IU; vitamin E, 60.0 mg; vitamin K, 3.0 mg; vitamin B_1_, 3.0 mg; vitamin B_2_, 8.0 mg; vitamin B_6_, 4.0 mg; vitamin B_12_, 0.02 mg; niacin 50.0 mg; pantothenic acid 15.0 mg; biotin 0.40 mg; folic acid 2.0 mg; Cu 15 mg; Fe 40 mg; Mn 100 mg; Zn 100 mg; I 1.0 mg; Se 1 mg.

**Table 3:** Growth performance of broiler chickens on day 21 and day 42^1)^

Item	Dietary treatment^2)^				SEM	p-value

	NC	PC	FBO	MBO		
Initial body weight (g)	47.4	46.8	46.7	47.1	0.154	0.710
Final body weight (g)	1,723^b^	1,897^a^	1,831^a^	1,881^a^	39.2	<0.010
Average daily feed intake (g/d)						
Day 1 to 21	49.6	51.0	50.6	51.2	0.360	0.738
Day 22 to 42	111	108	109	110	0.711	0.762
Day 1 to 42	80.4	79.4	79.7	80.4	0.240	0.944
Average daily gain (g/d)						
Day 1 to 21	31.5	34.8	33.4	34.1	0.699	0.059
Day 22 to 42	52.3	57.7	55.8	57.6	1.274	0.131
Day 1 to 42	41.9^b^	46.3^a^	44.6^a^	45.8^a^	0.983	<0.010
Feed conversion ratio						
Day 1 to 21	1.57^a^	1.47^b^	1.51^b^	1.50^b^	0.022	<0.010
Day 22 to 42	2.16	1.87	1.95	1.91	0.063	0.058
Day 1 to 42	1.92^a^	1.72^b^	1.79^ab^	1.75^b^	0.045	0.008
Mortality rate (%)	4.17	2.78	2.78	2.78	0.0347	0.862

SEM, standard error of the mean.

1) Data represent the mean of 6 pens (3 broilers/pen) per treatment.

2) NC, broiler fed a basal diet without additives; PC, broiler fed a basal diet with avilamycin at 10 ppm; FBO and MBO broiler fed a basal diet with unencapsulated basil oil and micro-encapsulated at 500 ppm.

a,b Different subscript letters indicate significant differences in the rows (p<0.05).

**Table 4:** Apparent ileal digestibility (AID) of broiler chickens on day 21 and day 42^1)^

Item	Dietary treatment^2)^				SEM	p-value

	NC	PC	FBO	MBO		
Day 21						
Dry matter	0.606^b^	0.657^a^	0.656^a^	0.675^a^	0.015	0.003
Crude protein	0.708^c^	0.762^ab^	0.739^bc^	0.778^a^	0.015	0.002
Ether extract	0.793^b^	0.835^ab^	0.828^ab^	0.846^a^	0.011	0.032
Crude fiber	0.592	0.646	0.610	0.658	0.015	0.171
Ash	0.315	0.354	0.346	0.342	0.009	0.677
Gross energy (MJ)	0.652^c^	0.674^bc^	0.691^ab^	0.706^a^	0.012	<0.001
Nitrogen free extract	0.582^b^	0.655^a^	0.650^a^	0.662^a^	0.019	0.006
Day 42						
Dry matter	0.619	0.664	0.646	0.658	0.010	0.055
Crude protein	0.688^b^	0.757^a^	0.734^ab^	0.758^a^	0.016	0.028
Ether extract	0.797^b^	0.854^a^	0.834^a^	0.847^a^	0.013	0.003
Crude fiber	0.591^b^	0.662^a^	0.649^a^	0.655^a^	0.016	0.002
Ash	0.282	0.348	0.303	0.308	0.014	0.107
Gross energy (MJ)	0.674^c^	0.696^bc^	0.703^ab^	0.731^a^	0.012	0.002
Nitrogen free extract	0.620	0.650	0.640	0.659	0.008	0.313

SEM, standard error of the mean.

1) Data represent the mean of 6 pens (3 broilers/pen) per treatment.

2) NC, broiler fed a basal diet without additives; PC, broiler fed a basal diet with avilamycin at 10 ppm; FBO and MBO broiler fed a basal diet with unencapsulated basil oil and micro-encapsulated at 500 ppm.

a–c Different subscript letters indicate significant differences in the rows (p<0.05).

**Table 5:** Jejunal histomorphology of broiler chickens on day 21 and day 42^1)^

Item	Dietary treatment^2)^				SEM	p-value

	NC	PC	FBO	MBO		
Day 21						
Villus height (VH, μm)	704^b^	714^b^	744^ab^	813^a^	24.6	0.036
Crypt depth (CD, μm)	117	115	113	111	1.43	0.469
Villus width (μm)	32.8	35.0	35.2	33.1	0.62	0.350
VH:CD	6.17^b^	6.31^b^	6.77^b^	7.77^a^	0.36	0.012
Villus surface area (mm^2^)	0.715^c^	0.768^b^	0.792^ab^	0.831^a^	0.020	<0.010
Day 42						
Villus height (VH, μm)	913^b^	923^b^	1,050^a^	1,127^a^	51.6	<0.010
Crypt depth (CD, μm)	140	142	148	150	2.22	0.197
Villus width (μm)	30.5	32.8	29.8	30.2	0.670	0.194
VH:CD	6.86^b^	6.67^b^	7.28^ab^	7.68^a^	0.220	0.022
Villus surface area (mm^2^)	0.864^c^	0.924^b^	0.973^b^	1.055^a^	0.040	<0.010

SEM, standard error of the mean.

1) Data represent the mean of 6 pens (3 broilers/pen) per treatment.

2) NC, broiler fed a basal diet without additives; PC, broiler fed a basal diet with avilamycin at 10 ppm; FBO and MBO broiler fed a basal diet with unencapsulated basil oil and micro-encapsulated at 500 ppm.

a–c Different subscript letters indicate significant differences in the rows (p<0.05).

**Table 6:** *Lactobacillus* spp. and *Escherichia coli* populations in ceco-colon of broiler chickens on day 21 and day 42^1)^

Item	Dietary treatment^2)^				SEM	p-value

	NC	PC	FBO	MBO		
Day 21						
*Lactobacillus* spp. (log_10_ cfu/g)	6.00	6.22	4.20	5.83	0.462	0.960
*Escherichia coli* (MPN/g)	9.27	11.00	11.00	9.93	0.426	0.236
Day 42						
*Lactobacillus* spp. (log_10_ cfu/g)	5.60	7.13	4.43	5.28	0.564	0.415
*Escherichia coli* (MPN/g)	5.47	3.14	2.01	3.62	0.721	0.471

SEM, standard error of the mean; MPN, most probable number.

1) Data represent the mean of 6 pens (3 broilers/pen) per treatment.

2) NC, broiler fed a basal diet without additives; PC, broiler fed a basal diet with avilamycin at 10 ppm; FBO and MBO broiler fed a basal diet with unencapsulated basil oil and micro-encapsulated at 500 ppm.

**Table 7:** Malondialdehyde (MDA) concentration and superoxide dismutase (SOD) activity in duodenal mucosa on day 21 and day 42^1)^

Item	Dietary treatment^2)^				SEM	p-value

	NC	PC	FBO	MBO		
Day 21						
SOD (U/mg protein)	155^c^	159^b^	166^ab^	174^a^	3.29	<0.001
MDA (nmol/mg protein)	5.00^a^	4.22^b^	4.06^b^	3.61^c^	0.265	<0.001
Day 42						
SOD (U/mg protein)	173^d^	181^c^	192^b^	200^a^	4.99	<0.001
MDA (nmol/mg protein)	7.06^a^	5.02^b^	4.84^b^	4.09^c^	0.71	<0.001

SEM, standard error of the mean; SOD, superoxide dismutase; MDA, malondialdehyde.

1) Data represent the mean of 6 pens (3 broilers/pen) per treatment.

2) NC, broiler fed a basal diet without additives; PC, broiler fed a basal diet with avilamycin at 10 ppm; FBO and MBO broiler fed a basal diet with unencapsulated basil oil and micro-encapsulated at 500 ppm.

a–d Different subscript letters indicate significant differences in the rows (p<0.05).

## SUPPLEMENTARY MATERIAL

**Supplementary Figure S1**. (A) Swelling percent (%), and (B) cumulative release (%) of MBO in SGF and SIF **Supplementary Figure S2**. The thermogravimetric analysis (TGA) thermograms of (A) FBO and (B) MBO **Supplementary Figure S3**. The structure of methyl chavicol and linalool as major constituents of basil oil (*Ocimum basilicum* Linn.) **Supplementary Table S1**. Antioxidant and antimicrobial activities of basil oil (*Ocimum basilicum* Linn.)
