# Supplementary material for: Microencapsulated basil oil (Ocimum basilicum Linn.) enhances growth performance, intestinal morphology, and antioxidant capacity of broiler chickens in the tropics
Source: Anim Biosci. 2022 Jan 3;35(5):752–62. doi: 10.5713/ab.21.0299 (PMC9065782; doi:10.5713/ab.21.0299)
Supplement: Supplementary file 1 [file ab-21-0299-suppl.pdf]

Appendix: Supplementary data for:

Microencapsulated basil oil (*Ocimum basilicum* Linn.) enhances growth performance, intestinal morphology, and antioxidant capacity of broiler chickens in the tropical region

Sureerat *Thuekeaw* [1], Kris *Angkanaporn* [2], Chackrit *Nuengjamnong* [1, 3, *]

[*] **Corresponding Author:** Chackrit Nuengjamnong

Tel.: +66 218-9684; **fax**: +66 251-2582, **E-mail address**: Chackrit.n@chula.ac.th

[1] Department of Animal Husbandry, Faculty of Veterinary Science, Chulalongkorn University, Bangkok 10330, Thailand

[2] Department of Veterinary Physiology, Faculty of Veterinary Science, Chulalongkorn University, Bangkok 10330, Thailand

[3] Food Risk Hub, Research Unit of Chulalongkorn University, Bangkok 10330, Thailand

ORCID

Sureerat Thuekeaw	https://orcid.org/0000-0002-7779-3593
Kris Angkanaporn	https://orcid.org/0000-0001-9195-136X
Chackrit Nuengjamnong	https://orcid.org/0000-0002-7883-4502

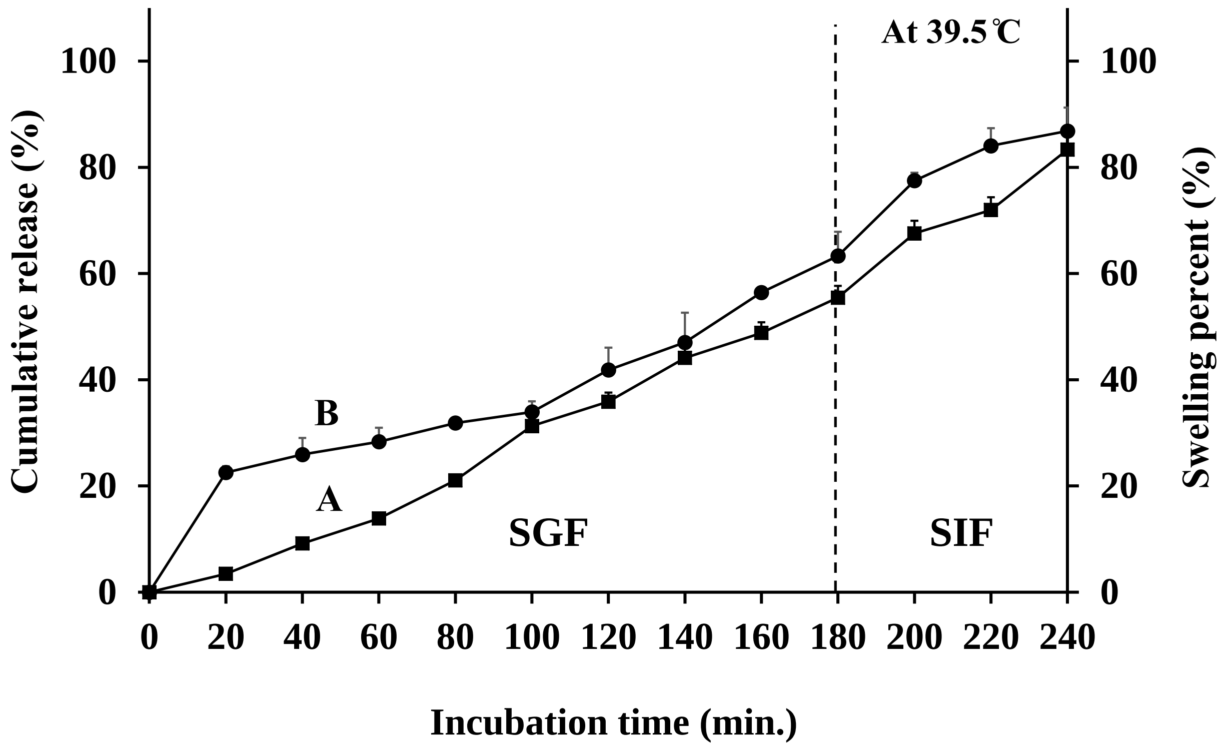

Appendix Fig. S1 (A) Swelling percent (%), and (B) cumulative release (%) of MBO in SGF and SIF at 39.5± 0.5 °C SGF; simulated gastric fluid and SIF; simulated intestinal fluid.

Swelling and cumulative release studies were carried out to evaluate the release performance of BO in the animal gut. A good correlation between swelling of MBO and release of BO can be clearly seen. The swelling was as high as 83.3% ± 3.3%, whereas the cumulative releases of BO was about 63.3% ± 4.6% and 86.8% ± 4.4% in SGF and SIF, respectively. Based on the release mechanism above, it is expected that microencapsulation leads to a successful release of BO at the lower part of intestine.

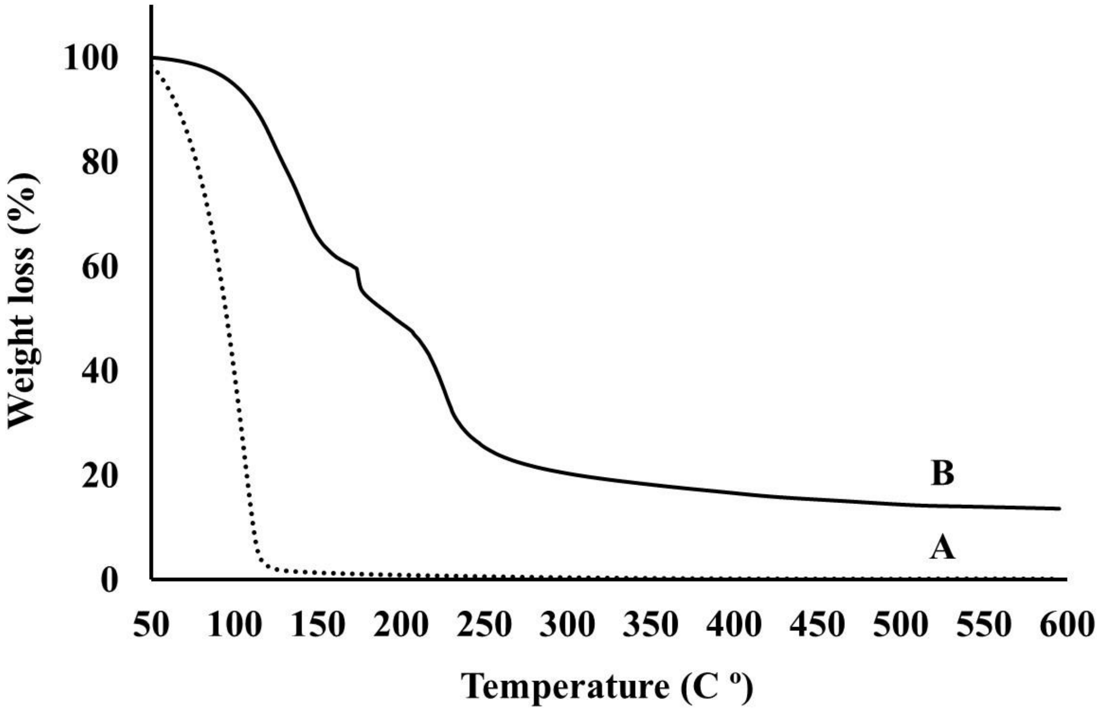

Appendix Fig. S2. The thermogravimetric analysis (TGA) thermograms of (A) FBO and (B) MBO

Thermal degradation behavior of FBO and MBO was comparatively studied. The decomposition of FBO was 95.62% of initial weight from 50 °C to 116 °C. In the case of MBO, the first degradation step from 100 °C to 149 °C for 40.3% is clarified. This might be due to corresponding to water and the degradation of BO. The weight loss from 160 °C to 220 °C, respectively was the decomposition of CS and SA. Based on TGA result, MBO represented a delay in the decomposition rate as compared to FBO

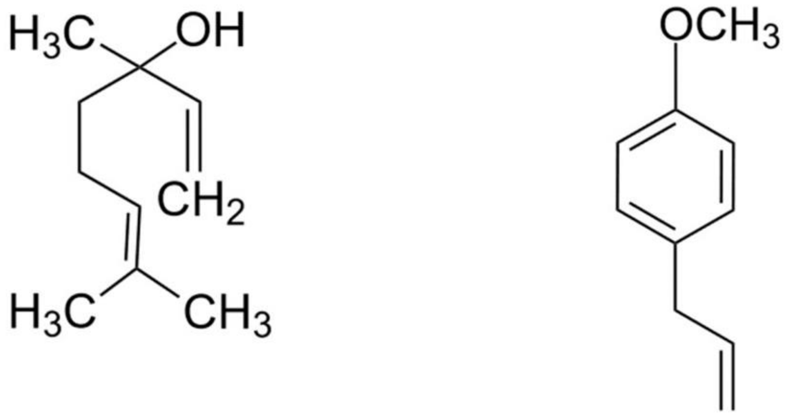

Methyl chavicol (Estragole) Linalool

Appendix Fig. S3. The structure of methyl chavicol and linalool as major constituents of basil oil (*Ocimum basilicum* Linn.)

Appendix Table S1 Antioxidant and antimicrobial activities of basil oil (*Ocimum basilicum* Linn.)

Sample	DPPH radical scavenging (%)	Inhibition zone diameter (mm.)		
		E. coil	*S. aureus*	*Salmonella* spp.
Basil oil	66.2 ± 0.7	11.0 ± 1.0	16.7 ± 1.5	10.0 ± 1.0

Antioxidation and antimicrobial abilities of basil oil as evaluated by DPPH radical scavenging and by inhibition zone, respectively.

The scavenging activity of BO (100 mg/mL) was found to be 66.2% ± 0.8% and the IC_{50} was 72.8 µg/mL ± 1.8 µg/mL. The antibacterial activity of BO (400 mg/mL) as evaluated from the inhibition zones were found to be 16.7 mm ± 1.5 mm, 11.0 mm ± 0.5 mm and 10.0 mm ± 1.0 mm for *S. aureus*, *E. coli*, and *Salmonella* spp., respectively.
